# Radiological manifestations of chondromyxoid fibroma in the zygoma: A case report and literature review

**DOI:** 10.1259/bjrcr.20210008

**Published:** 2021-05-12

**Authors:** Atefeh Zeinoddini, Amy Bezold, Obadah Ezzeldin, Huda AL Jadiry

**Affiliations:** 1 Neuroradiology Division, Department of Radiology, University of Texas Medical Branch, Galveston, TX, USA

## Abstract

Chondromyxoid fibroma (CMF) is a rare benign bone tumor of cartilaginous origin, with an extremely rare craniofacial occurrence. Considering its rarity, craniofacial CMF presents a diagnostic challenge for radiologists. To our knowledge, only seven cases of zygomatic CMF have been described in the literature, only one of which was in the paediatric age group. Furthermore, none of the currently reported cases include MRI findings of zygomatic CMF. Here, we present a paediatric case of CMF of the zygoma with a comprehensive literature review of the reported cases, focusing on their radiological features and its differential diagnosis.

## Introduction

Chondromyxoid fibroma (CMF) is a rare, benign bone tumour of cartilaginous origin which accounts for <1% of primary bone tumours.^1^ CMF was first described by Jaffe and Lichtenstein in 1948 as a bony tumour with a benign clinical course that can be commonly mistaken as chondrosarcoma.^2^ CMF most frequently affects the proximal tibia and the distal femur and usually occurs in the second and third decades of life. Craniofacial occurrence of the CMF is extremely rare, reported to account for 5.4% of total CMF cases.^3^ Radiological manifestations of CMF are nonspecific and considering its rarity, craniofacial CMF presents a diagnostic challenge for radiologists. To our knowledge, only seven cases of zygomatic CMF have been described in the literature, and none of these have presented the MRI findings of the zygomatic CMF. Also, only one case has been reported in the paediatric age group. Here, we present a paediatric case of CMF of the zygoma, focusing on the radiological features of CMF and its differential diagnosis. To our knowledge, this is the first report that describes the MRI findings of CMF of the zygoma and focuses on radiological findings of this rare presentation.

## Case presentation

### Clinical presentation

An 8-year-old girl presented to her paediatrician complaining of atraumatic ‘bump’ on her right cheek for two weeks. Examination revealed a non-tender firm nodule on the right zygomatic arch without cutaneous abnormalities.

### Investigation

**Figure 1.**
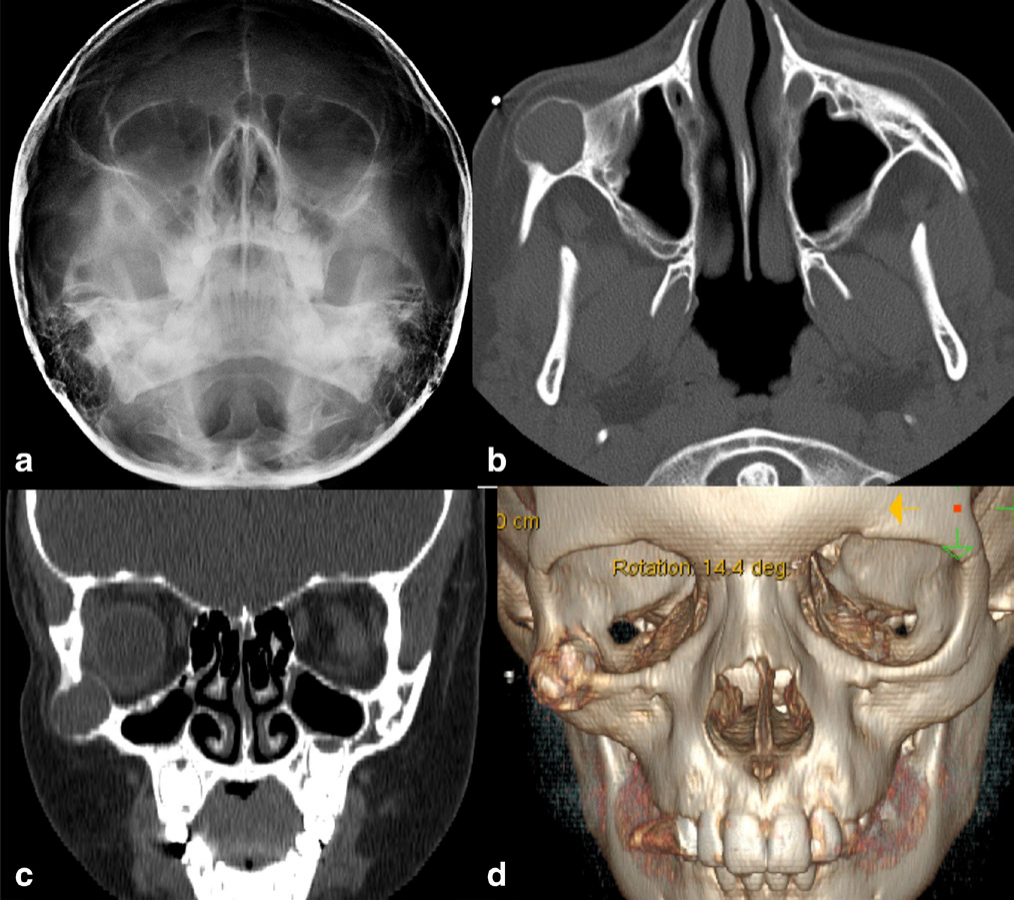
(**a**) Plain facial radiograph showing sclerotic lesion with central lucency in the right zygomatic bone. Non-enhanced CT (**b**) axial, and (**c**) coronal images demonstrate a well-demarcated round, expansile lucent lesion in the right zygomatic bone with homogeneous internal attenuation (53 hounsfield unit (HU)) close to that of a normal muscle (35 to 55 HU). (**d**) 3D reformat of the of the lesion.

Plain facial bone radiographs showed a sclerotic lesion with central lucency in the right zygomatic bone (Figure 1a). A subsequent non-contrast maxillofacial computerized tomography (CT) revealed a well-defined, round, expansile, soft-tissue density lesion arising from the right zygomatic bone, measuring 1.7 × 1.6 × 1.6 cm. The lesion was surrounded by variably thinned cortex with a narrow zone of transition to normal bone. Scalloping of the zygoma and bulging of the expanded zygoma into the orbit were seen. There was no evidence of erosion or invasion of the adjacent soft tissues (Figure 1b, c and d).

**Figure 2.**
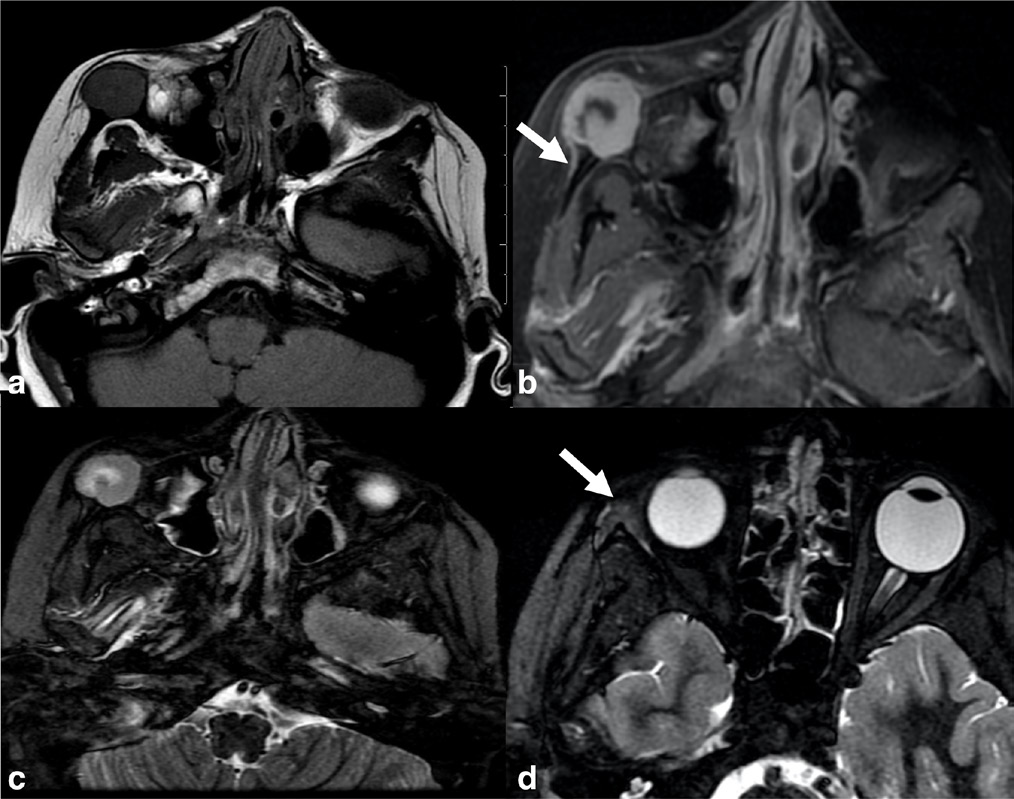
(**a**) Axial T1 sequence showed a homogeneously hypointense to isointense expansile, lobulated lesion with distinct borders. (**b**) On axial post-contrast, fat-saturated T1 sequence, the lesion illustrates solid peripheral enhancement with central non-enhancing component. The arrow shows a thin layer of periosteal thickening and enhancement. (**c**) Axial STIR sequence, the lesion shows intermediate signal with a curvilinear focus of hyperintense signal centrally. (**d**) Axial STIR sequence at a higher level demonstrates a focal area of hyperintense signal at the orbital process of the zygoma and anterior aspect of the lateral orbital wall likely representing bone marrow oedema (arrow).

To further characterize the lesion, facial magnetic resonance imaging (MRI) with and without contrast was performed and showed, compared to adjacent muscle, a homogeneously hypointense to isointense expansile, lobulated lesion with distinct borders on T1 sequence (Figure 2a). On the post-contrast fat-suppressed T1 sequence, the lesion showed avid enhancement with a curvilinear focus of non-enhancement centrally (Figure 2b). Adjacent periosteal thickening and enhancement in the lateral orbital wall, as well as along the anterior maxilla were seen (Figure 2b, white arrow). The lesion showed intermediate signal on short-TI inversion recovery (STIR) with a curvilinear focus of hyperintense signal centrally (Figure 2c). A focal area of hyperintense signal in STIR sequence at the orbital process of the zygoma and anterior aspect of the lateral orbital wall likely represent bone marrow oedema (Figure 2d, white arrow). DWI signal was heterogeneous with areas of T2 shine-through but no areas of diffusion restriction.

**Figure 3.**
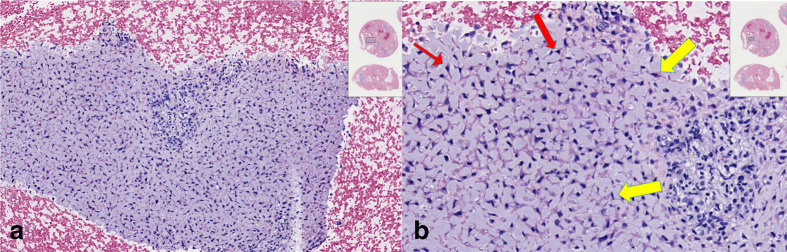
Surgical pathology slides reveal fragments of hypo- and hypercellular areas. (**a**) Pale-staining myxoid background underlying benign appearing cells with indistinct cytoplasmic borders. (**b**) Scant isolated and small clusters of spindle-shaped tumour cells (yellow arrows) and stellate-shaped cells (red arrows).

A core biopsy of the lesion was performed which showed features of spindle cell neoplasm, favouring CMF. Conservative surgical curettage without osseous resection was performed. Surgical pathology confirmed diagnosis of CMF (Figure 3).

**Figure 4.**
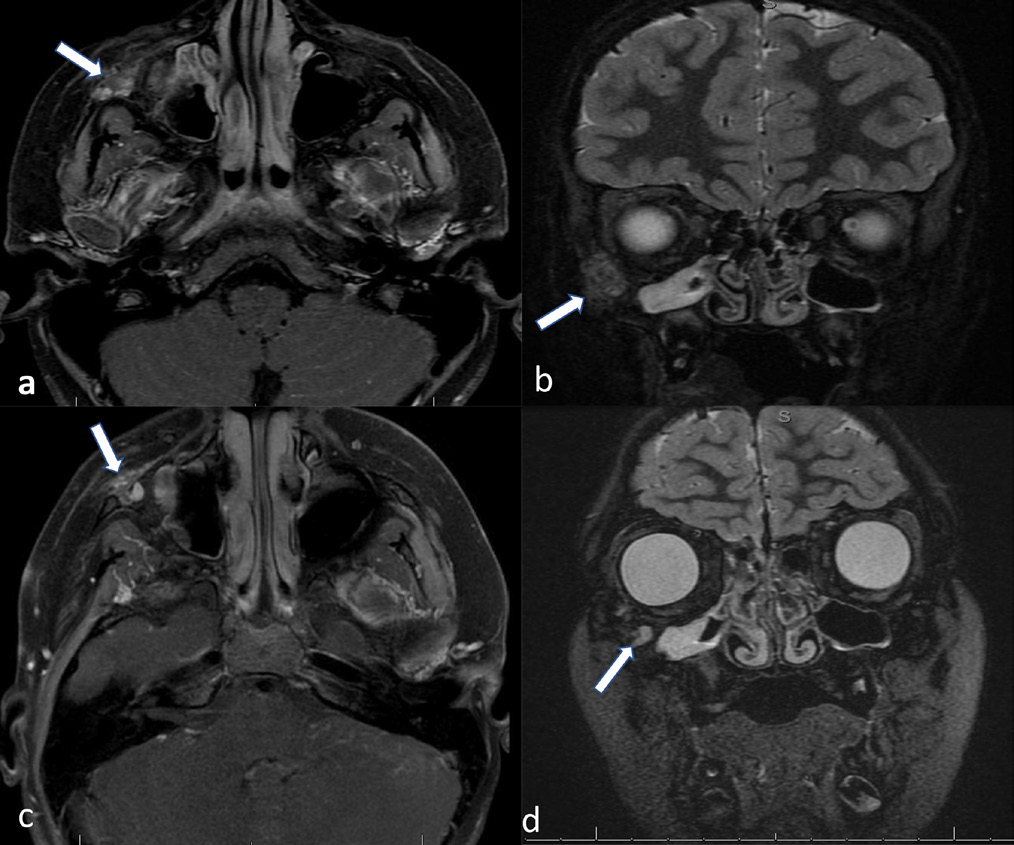
(**a**) Axial T1 fat-saturated-enhanced sequence and (**b**) coronal STIR sequence four months post-resection showed interval resection of the previously demonstrated expansile lesion centred in the right zygomatic bone. There was, however, a mildly STIR hyperintense signal with heterogeneous enhancement that could represent residual tumour versus granulation tissue. (**c**) Axial T1 fat-saturated-enhanced sequence and, (**d**) coronal STIR sequence 12 months post-resection demonstrate enlargement of a discrete sub-centimeter focus of enhancement at the site of the resection, with signal characteristics similar to the original mass lesion. These findings are suspicious for tumour residuum/recurrence.

Post-operative MRI at month four and twelve after resection both revealed evidence of possible residual tumour or recurrence (Figure 4).

## Literature review and discussion

The occurrence of isolated CMF in the zygoma is extremely rare. A literature review was conducted in PubMed and Scopus, using the combination of the following key terms: chondromyxoid fibroma and zygoma or skull. We identified seven published cases of CMF arising from the zygoma summarized in Table 1.^4–10^ This summary includes patient demographics and clinical presentation, lesion location and radiological findings, as well as surgical management, complications and evidence of lesion recurrence after treatment.

**Table 1.** Summary of reported case of patients of chondromyxoid fibroma (CMF) of zygoma, including patients’ radiological characteristics

Study	Age (yr), Gender	Clinical symptoms, Onset of symptoms	Size, Side	Radiological Findings	Management	Outcomes
Ashraf et al. 2017^4^	47, Female	Painful swelling of left side of face, 15 years prior to presentation	3 × 2 cm, Left	Radiograph: a destructive mass in zygoma CT: a well-demarcated lobulated expansible osteolytic lesion, with partial destruction of the cortical Zygomatic bone	En bloc resection	No recurrence in 2 year follow up
Bucci et al, 2005^5^	51, Male	Painless swelling of the right face overzygomatic bone, 6 months	3 × 3 cm, Right	CT: well-demarcated lobulated expansible osteolytic lesion in the Zygomatic body, with partial destruction of the lateral wall of the orbit	En bloc resection	No recurrence in 2 year follow up with CT scan.
Carr et al, 1992^6^	41, Female	Asymptomatic lump over her cheek, 8 month	3 cm, Left	CT: A lesion with soft tissue attenuation at the zygomatic body and anterior one third of zygomatic arch, extending to the orbit wall	En bloc resection.	No recurrence in follow-up CT in 18 months.
Chen et al. 2008^7^	-, -	-	-	-	En bloc resection.	No recurrence in follow up by CT for at least 12 months.
Pintor et al.^8^	68, Female	Swelling over the left zygomatic region, with intermittent pain, NM	3 × 3 cm, Left	CT: A lytic expansile lesion	En bloc resection.	No recurrence in follow-up CT in 6 months.
Sudhakara et al. 2018^9^	3.5, Male	Asymptomatic swelling over the region of left zygomatic arch, 6 months.	2 × 1 cm, left	CT: a bony lesion in the zygomatic arch	En bloc resection	No clinical symptoms of recurrence in 14 month follow-up.
Zhu et al. 2018^10^	30, Male	Pain in the region of left zygoma, 1 year	Not provided, Left	Not provided	Conservative curettage	Recurrence in 6 months with pain and swelling over surgical field

The median age of reported patients with CMF of the zygoma, including our patient was 41 years old, ranging from 3.5 to 68 years old. Four patients were female, and three patients were male. CMF affected the left side in five patients and right side in two patients. The lesions ranged in maximal dimension from 1.7 to 3 cm, with the majority of lesions in the case reports being detected at 3 cm (median lesion size 3 cm). Patients presented with various clinical presentations, including painless swelling in four cases and painful swelling in three others.

In part due to heterogeneous imaging findings and its rarity in non-axial bones, CMF is often misdiagnosed. Zillmer et al reported a case series of 36 patients with CMF: at initial presentation, 67% of patients received the correct diagnosis, 22% of the patients were misdiagnosed, and 11% of the patients had no diagnosis.^11^

On plain radiograph, CMF of the appendicular and axial skeleton appears as a well-defined, lobulated or oval eccentric lytic lesion with geographic bone destruction. Sclerotic margins can be seen in about 80% of patients, and septation (pseudo trabeculation) is seen in 60% of patients. Internal matrix calcification is an uncommon radiographical finding, seen in 2–15% of lesions. Periosteal reaction has been reported in 50% of the patients.^1,3,12,13^

Our case is unique compared to the others in current literature, in that we provide MRI characterization of zygomatic CMF. MRI is used to evaluate the relationship and possible extension of the tumour to adjacent structures and to further characterize a lesion that may not have a clear diagnosis after radiograph or CT imaging. Kim et al retrospectively assessed the MRI features of CMF in 19 histopathologically confirmed CMF and found several similar features, including low-to-intermediate signal in T1W sequences and intermediate-to-high signal in T2W sequences (when compared to adjacent muscle). Post-contrast images showed peripheral enhancement with a central non-enhancing portion.^14^ While these features were identified mostly in CMFs found in long bones, most of the same features are identified in our case of CMF in the zygoma as well. Differential diagnosis of CMF and their radiological manifestations are summarized in Table 2.

**Table 2.** Radiological features of chondromyxoid fibroma (CMF) and its differential diagnosis

	Radiograph and CT	MRI
Chondromyxoid fibroma^1,3,14^	Lytic expansile lesion Well-defined margin Lobulated or oval eccentric lesion Geographic bone destruction Septation/pseudotrabeculation No cortical destruction No soft tissue component Rarely extend through the cortex	T1: low-to-intermediate signal T2: intermediate-to-high signal T1 contrast (Gadolinium): Peripheral nodular enhancement in about 70% of cases Diffuse heterogeneous or homogeneous enhancement in 30% Low signal can be seen in calcified area and sclerotic margin in all sequences
Chondrosarcoma	50% lytic Cortical destruction Soft tissue mass Matrix calcifications: rings and arc or popcorn calcifications Moth eaten appearance Periosteal reaction Pathological fracture Intramural matrix mineralization Endosteal scalloping, affecting > two-third of the cortical thickness Heterogeneous enhancement in CT with contrast	T1: low-to-intermediate signal T2: variable signal intensity T1*c* + Gadolinium: Heterogeneous moderate to intense enhancement Enhancement can be in the periphery (rim-like) and/or the trans-lesional septae
Chondroblastoma	Lytic lesion well-defined Smooth or lobulated May have a thin sclerotic rim Internal calcification can be seen Solid periosteal reaction may present Endosteal scalloping may be seen Rarely extend through the cortex	T1: intermediate signal T2: variable signal intensity T1*c* + Gadolinium: Heterogeneous moderate enhancement, surrounding bone and soft tissue oedema will enhance Fluid-fluid level may present.
Chondroma^12^	Usually purely lytic Small, usually <5 cm lesions Geographic lytic lesion Could be expansible Endosteal scalloping may present (affects less than two-third of the cortical thickness) Well-defined margin Calcifications may present as, popcorn like calcifications, ring and arc calcifications No aggressive behaviour No periosteal reaction No cortical destruction No soft tissue component Rarely extend through the cortex	Well-defined margin Lobulated margin T1: low-to-intermediate signal Calcifications will have low signal T2: High signal intensity T1*c* + Gadolinium: variable enhancement pattern, enhancement could be seen in the periphery and/or the trans-lesional septae Focal foci of signal drop out can be seen at calcified regions, calcified chondroid will have low signal in all MRI sequences

Although MRI can help further characterize the lesion and suggest CMF as a possible diagnosis, it is noteworthy that definite diagnosis cannot be made based on radiological features alone, and biopsy is necessary for definitive diagnosis. Histopathologically, CMF has been defined by the World Health Organization (WHO) as “a benign tumour characterized by lobulated areas of spindle-shaped or stellate cells with abundant myxoid or chondroid intercellular material separated by zones of more cellular tissue rich in spindle-shaped or round cells with varying number of multinucleated giant cells of different sizes.”^15^ Our case had the same histological features but with relatively greater mitosis.

Surgical resection of the CMF is the treatment of choice. Surgical options vary from conservative curettage to en bloc resection. Recurrence rate following curettage of CMF tumour reported to vary from 20–80%, therefore en bloc resection is usually recommended, especially in children who are more prone to tumour recurrence. Radiation therapy is not recommended, since it has been reported to be associated with development of chondrosarcoma.^3^ In our literature review of the seven described cases, six patients underwent en bloc resection and one patient underwent curettage. Recurrence did not occur in any of the patients who underwent en bloc resection. However, recurrence occurred in the patient who underwent curettage. Given the high recurrence rate, follow-up is recommended. We do recommend baseline MRI prior to the surgery and follow-up MRI to assess for subtle recurrence changes that might be overlooked by CT scan. Our protocol for these lesions includes the following sequences: T2 and STIR in axial and coronal planes, T1 fat saturated MRI pre-contrast and post-contrast in all planes.

One of the limitations of the presented literature review is that the focus of previous case reports was not radiological manifestation of CMF, which limits the evaluation of the radiological features of CMF of the zygoma on prior cases.

## Conclusion

CMF is rare and can be encountered outside of the classical locations, including very rarely in the craniofacial bones. CMF has quite variable and non-specific imaging finding, but when found outside normal locations it appears to broadly follow imaging characteristics of CMF in more common locations. Definitive diagnosis is only possible via histopathology. Ongoing imaging surveillance, ideally with MRI, will be helpful due to the locally aggressive nature of the disease and high likelihood of recurrence. Adding more cases to the literature will enhance radiologists’ awareness of the possibility of this pathology when presenting in uncommon locations.

## Learning points

- Chondromyxoid fibroma (CMF) in the facial bones is rare but when reported has similar imaging characteristics to CMF in the more common locations such as the long bones.
- The major roles of imaging in CMF are to determine lesion extension into adjacent structures, to evaluate for residual disease post resection or curettage, and to monitor for disease recurrence. Definitive diagnosis is ultimately accomplished by histopathology.
- CMF is a benign bone tumour, but can have aggressive behaviour with high recurrence rate. En bloc resection is favoured over curettage to decrease likelihood of recurrence.
